# Glucagon-like peptide-1 receptor agonist-induced cholecystitis and cholelithiasis: a real-world pharmacovigilance analysis using the FAERS database

**DOI:** 10.3389/fphar.2025.1557691

**Published:** 2025-07-08

**Authors:** Chao Tao, Yinhui Zhang, Tenggang Wan, Wenting Zhao, Jing Chen, Ke Wang, Liuxuan Yang, Guojun Wang, Qian Ding, Jinlu Shang, Meiling Zhou

**Affiliations:** ^1^ Department of Pharmacy, The Affiliated Hospital, Southwest Medical University, Luzhou, China; ^2^ Department of Clinical Pharmacy, School of Pharmacy, Southwest Medical University, Luzhou, China; ^3^ Department of Rehabilitation, The Affiliated Hospital of Southwest Medical University, Luzhou, China; ^4^ Department of Clinical Pharmacy, The Third Hospital of Mianyang, Sichuan Mental Health Center, Mianyang, China; ^5^ Department of Pharmacy, West China Hospital Sichuan University Jintang Hospital, Chengdu, China

**Keywords:** glucagon-like peptide-1 receptor agonists, cholecystitis, cholelithiasis, disproportionality analysis, pharmacovigilance, FAERS

## Abstract

**Background:**

With the widespread use of glucagon-like peptide-1 receptor agonists (GLP-1 RAs) in managing diabetes and obesity, the occurrence of GLP-1 RA-induced cholecystitis and cholelithiasis has raised increasing concern among healthcare professionals.

**Methods:**

This study extracted adverse event reports of GLP-1 RA-induced cholecystitis and cholelithiasis from the FDA Adverse Event Reporting System database, covering Q1 2004 to Q2 2024. Disproportionality analysis methods, including the reporting odds ratio, proportional reporting ratio, and Bayesian confidence propagation neural network, were employed to identify associations between GLP-1 RAs and these AEs. The analysis focused on the five most commonly prescribed GLP-1 RAs, evaluated at both high-level term and preferred term levels.

**Results:**

A total of 1,829 reports were identified in which GLP-1 RAs were listed as the primary suspect drug, involving 1,651 patients. All three signal detection methods indicated a positive signal between GLP-1 RAs and these conditions. The majority of cases occurred in patients aged 45 years and older, with a significantly higher prevalence in females. The median onset time of GLP-1 RA-induced cholecystitis and cholelithiasis was 182 days, with variations observed across different drugs, genders, and age groups.

**Conclusion:**

This study provides a comprehensive pharmacovigilance analysis of GLP-1 RA-induced cholecystitis and cholelithiasis, offering valuable insights into the prevention and management of these AEs.

## 1 Introduction

Diabetes mellitus is a metabolic disorder characterized by chronic hyperglycemia, resulting from defects in insulin secretion and/or insulin resistance. According to recent data, the global prevalence of diabetes reached 529 million individuals by 2021, with type 2 diabetes mellitus (T2DM) accounting for 96.0% of all cases, particularly reflecting its high prevalence among older adults (Ong et al., 2023). Projections indicate that the number of individuals with diabetes worldwide will rise to 783.2 million by 2045 (Sun et al., 2022). Diabetes not only severely diminishes the quality of life but also leads to various complications, including cardiovascular diseases, nephropathy, retinopathy, and neuropathy, which can result in severe disability or even death (Wei et al., 2021; Yun and Ko, 2021). Moreover, obesity has become a global health crisis, contributing to the deaths of 2.4 million women and 2.3 million men, as well as 70.7 million disability-adjusted life years (DALYs) in women and 77.0 million DALYs in men as of 2017 (Dai et al., 2020). Between 2000 and 2019, the annual growth rate of obesity-related mortality was 0.48%, and it is projected that the prevalence of obesity will increase by 39.8% from 2020 to 2030 (Chong et al., 2023).

In this context, glucagon-like peptide-1 receptor agonists (GLP-1 RAs), a novel class of antidiabetic and anti-obesity agents, have gained widespread recognition for their safety, efficacy, and metabolic benefits in managing T2DM and obesity. GLP-1 RAs exert their effects through multiple mechanisms, including promoting insulin secretion, suppressing glucagon release, enhancing pancreatic beta-cell function, reducing appetite, and delaying gastric emptying (Nauck et al., 2021). These actions effectively lower blood glucose levels and reduce body weight. Furthermore, GLP-1 RAs have demonstrated efficacy in providing cardiovascular and renal protection (Trujillo et al., 2021; Ansari et al., 2024). However, with the expansion of GLP-1 RA usage, the number of AE reports associated with these agents has also increased, raising growing concerns among healthcare professionals (Watanabe et al., 2024).

Drug-induced cholecystitis and cholelithiasis refer to gallstone formation and gallbladder inflammation caused by prolonged or inappropriate medication use, constituting common adverse reactions within the hepatobiliary system. Recent studies have identified cholecystitis as an AE associated with GLP-1 RAs (Wu et al., 2022). Additionally, case reports have documented the occurrence of cholelithiasis in obese patients treated with GLP-1 RAs (Moll et al., 2024). These conditions can significantly impact disease progression and prognosis during the treatment of T2DM and obesity. However, systematic investigations leveraging the FDA Adverse Event Reporting System (FAERS) database to explore GLP-1 RA-induced cholecystitis and cholelithiasis remain limited. Furthermore, there is a notable lack of comparative analyses across different GLP-1 RAs and detailed assessments of the time to onset of these AEs in affected patients (Liang et al., 2024).

This study conducted a comprehensive pharmacovigilance analysis of GLP-1 RA-induced cholecystitis and cholelithiasis using real-world data. We systematically screened data from the FAERS database covering from Q1 2004 to Q2 2024, specifically focusing on reports of cholecystitis and cholelithiasis where GLP-1 RAs were identified as the primary suspect drug. Data mining algorithms were employed to identify potential signals between GLP-1 RAs and these AEs. Disproportionality analysis was performed at both high-level term (HLT) and preferred term (PT) levels to explore the strength of associations and evaluate potential drug-related risks. Furthermore, comparative analyses of cholecystitis and cholelithiasis induced by the five most commonly prescribed GLP-1 RAs, including the onset time of these AEs, were carried out. Through this research, we aim to provide a deeper understanding of GLP-1 RA-induced cholecystitis and cholelithiasis, which will assist in their prevention and management. The findings will offer clinicians valuable insights into the safety profiles of GLP-1 RAs and provide evidence for drug selection. Moreover, this study contributes to improving the rational use of pharmacotherapy, enhancing patient safety.

## 2 Methods

### 2.1 Data source

This study utilized the FAERS database to perform a comprehensive pharmacovigilance analysis of AEs related to cholecystitis and cholelithiasis. The FAERS database is a publicly accessible, voluntary reporting system that compiles safety reports on approved drugs and therapeutic biologics submitted by healthcare professionals, pharmaceutical manufacturers, patients, and others. Since 2004, FAERS has provided public access to its data, which is quarterly updates. The database comprises seven datasets: demographics and administrative information (DEMO), drug information (DRUG), adverse event details (REAC), patient outcomes (OUTC), report sources (RPSR), therapy start and end dates (THER), and indication/diagnosis (INDI).

**FIGURE 1:**
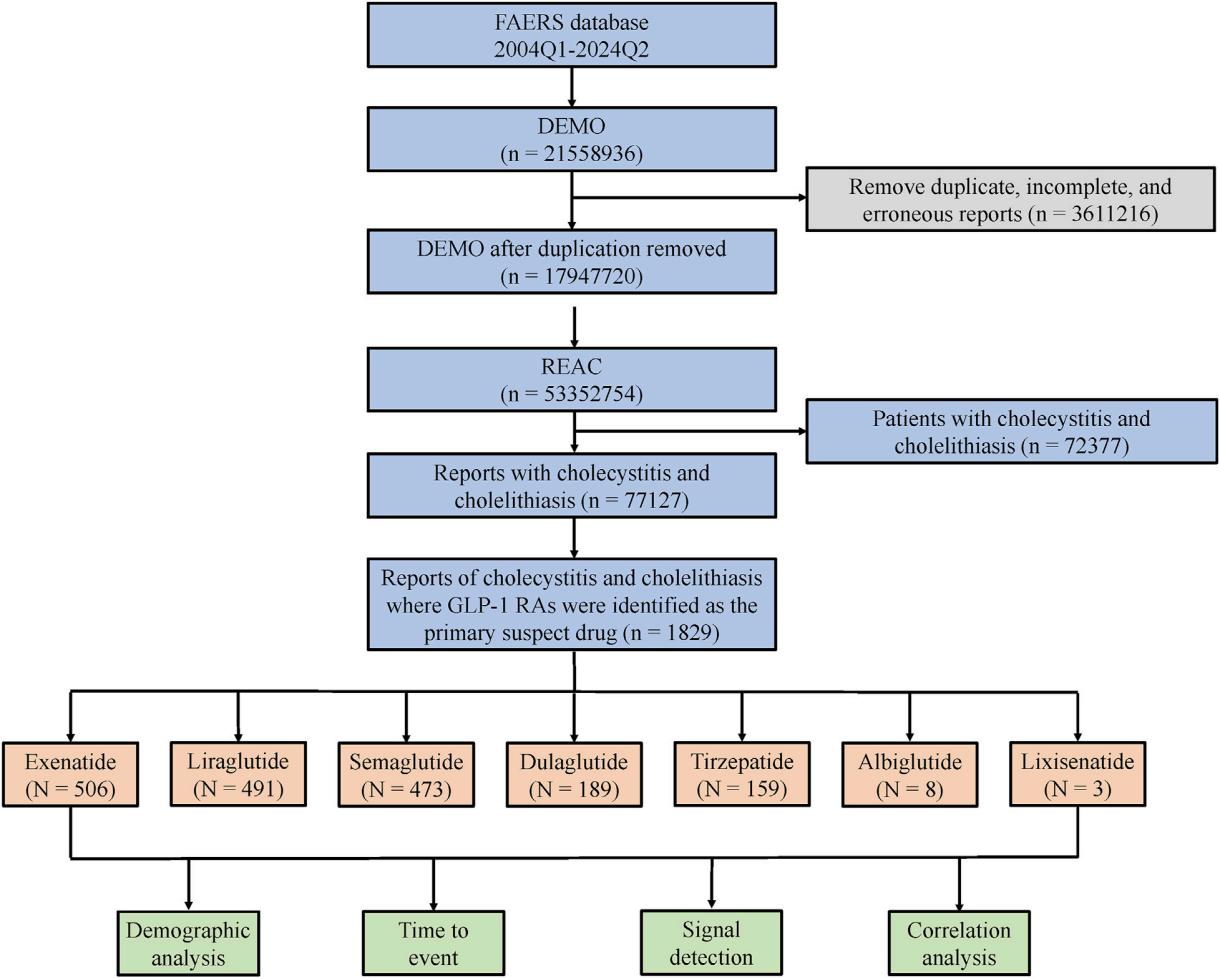
The flow chart of the study.

For this study, data from Q1 2004 to Q2 2024 were extracted. The analysis process is outlined in the flowchart presented in Figure 1. Following FDA guidelines and official recommendations, we utilized SAS 9.4 software for data screening and processing. Duplicate, incomplete, and erroneous reports were excluded to ensure the accuracy and consistency of the analysis. Only records of cholecystitis and cholelithiasis where GLP-1 RAs were listed as the primary suspect drug were included. Due to the limited number of reports for albiglutide and lixisenatide, which were insufficient to allow a comprehensive evaluation, this study focused on the remaining GLP-1 RAs, including exenatide, liraglutide, semaglutide, dulaglutide, and tirzepatide.

### 2.2 Identification of relevant reports

In this study, AEs were coded using PTs from version 26.1 of the Medical Dictionary for Regulatory Activities (MedDRA), which provides standardized descriptions for specific medical conditions. Specific PT can be matched with multiple HLTs, high-level group terms (HLGTs), and system organ classes (SOCs). To ensure specificity and accuracy in identifying relevant AE reports, this study referenced the “cholecystitis and cholelithiasis (HLT)” entry in MedDRA version 26.1. Data mining and report recognition were carried out within this HLT. The detailed PTs used for identification are listed in Table 1.

**TABLE 1:** PTs included in the “cholecystitis and cholelithiasis (HLT)” entry.

MedDRA code	PT
10008629	Cholelithiasis
10008612	Cholecystitis
10008617	Cholecystitis chronic
10008614	Cholecystitis acute
10062631	Cholecystitis infective
10008630	Cholelithiasis obstructive
10066884	Pseudocholelithiasis
10082088	Haemorrhagic cholecystitis
10056668	Emphysematous cholecystitis
10017649	Gallstone ileus
10068884	Cholelithiasis migration
10084002	Ischaemic cholecystitis
10088969	Eosinophilic cholecystitis

### 2.3 Analytical methods

This study employed descriptive analysis to comprehensively summarize the clinical characteristics of patients experiencing GLP-1 RA-induced cholecystitis and cholelithiasis. The analysis included factors such as the year of reporting, patient age, gender, reporter type, reporting country, and patient outcomes. To further investigate, data mining algorithms were applied for disproportionality analysis to quantitatively detect AE signals in the pharmacovigilance database. A classic 2 × 2 contingency table (Table 2) was used to compare the occurrence frequency of AEs linked to the specific GLP-1 RAs with background frequency to establish statistical associations.

**TABLE 2:** The classic two-by-two contingency table.

Drug types	Number of reports for target AE	Number of reports for other AEs	Total
Target drug	a	b	a + b
Other drugs	c	d	c + d
Total	a + c	b + d	a + b + c + d

The potential AE risks were evaluated using three data mining algorithms: the reporting odds ratio (ROR), proportional reporting ratio (PRR), and Bayesian confidence propagation neural network (BCPNN). The formulas and evaluation criteria for these methods are detailed in Table 3. The ROR, PRR, and information component (IC) values serve as indicators to compare AE risks related to different drugs. A signal is considered positive when the corresponding values meet the predefined thresholds. Higher values reflect stronger associations between a drug and the AE, suggesting an increased risk of cholecystitis and cholelithiasis. The onset time of drug-induced cholecystitis and cholelithiasis was calculated based on the difference between the date of the first reported AE and the start date of the primary suspected medication. The median onset times of GLP-1 RA-induced cholecystitis and cholelithiasis were statistically analyzed and compared.

**TABLE 3:** Overview of algorithms utilized for signal detection.

Algorithms	Formulas	Criteria
ROR	$\text{ROR}=\frac{\text{ad}}{\text{bc}}$	Lower limit of 95% CI > 1, a ≥3
	$95\%\text{ CI}={\;e}^{{\ln\left(\text{ROR}\right)\;\pm \;1.96\;\left(\frac{1}{a}+\frac{1}{b}+\frac{1}{c}+\frac{1}{d}\right)}^{0.5}}$	
PRR	$\text{PRR}=\frac{a/\left(a+b\right)}{c/\left(c+d\right)}$	PRR ≥2, χ^2^ ≥ 4, a ≥3
	$\chi^{2}=\frac{{\left(\text{ad}\;‐\;\text{bc}\right)}^{2}\left(a+b+c+d\right)}{\left(a+b\right)\left(c+d\right)\left(a+c\right)\left(b+d\right)}$	
BCPNN	${\text{IC}=\log}_{2}\frac{a\left(a+b+c+d\right)}{\left(a+c\right)\left(a+b\right)}$	IC025 > 0
	$\text{IC}025=E\left(\text{IC}\right)‐2{\left[V\left(\text{IC}\right)\right]}^{0.5}$	

CI, confidence interval; χ^2^, chi-squared; IC, information component; E(IC), the IC, expectations; V(IC), the variance of IC.

## 3 Results

### 3.1 Descriptive analysis

**FIGURE 2:**
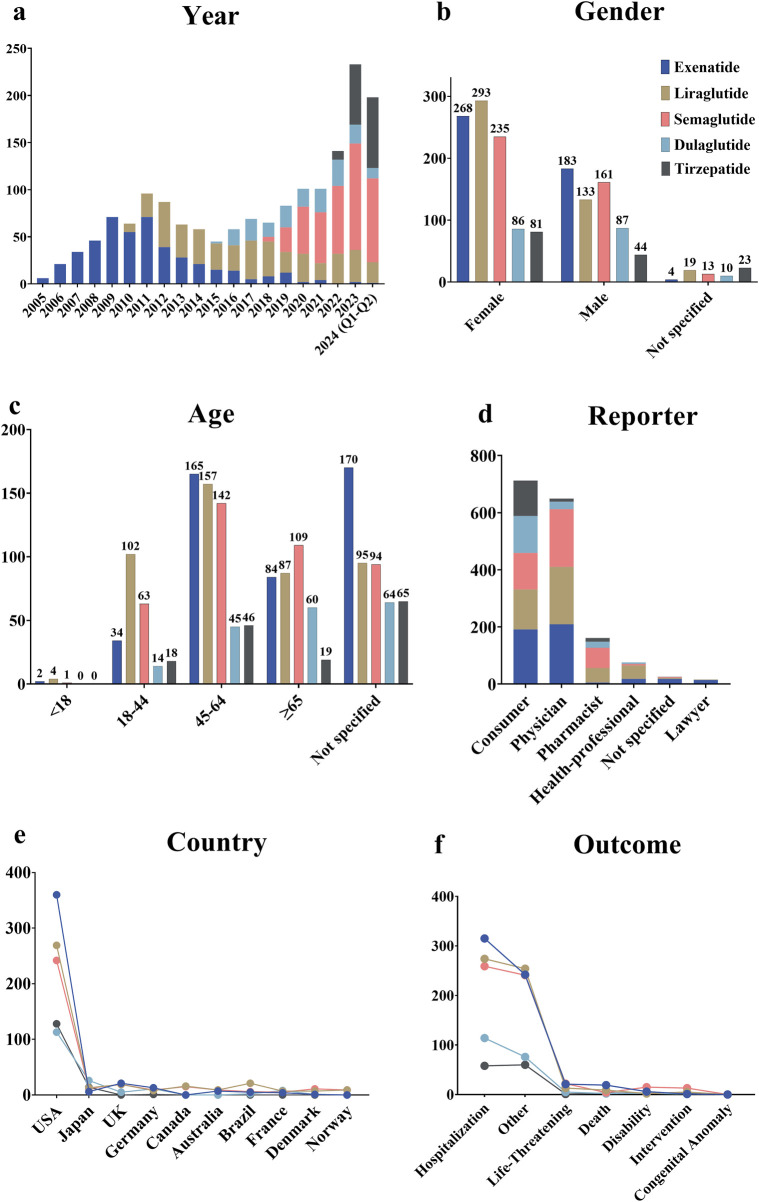
Overview of AE reports related to cholecystitis and cholelithiasis in the FAERS database. **(a)** Annual number of AE reports. **(b)** Gender distribution of patients. **(c)** Age distribution of patients. **(d)** Occupational distribution of reporters. **(e)** Top 10 countries by the number of reports. **(f)** Outcome distribution of AEs in patients.

The results revealed that from Q1 2004 to Q2 2024, a total of 1,651 patients treated with GLP-1 RAs were included in the FAERS database related to cholecystitis and cholelithiasis. In total, 1,829 AE reports were identified through a search of the “cholecystitis and cholelithiasis (HLT)” entry. Since the launch of the first GLP-1 RA, exenatide, in 2005, the number of reports has exhibited an overall upward trend, which corresponds with the market launch of subsequent drugs in this class. For example, liraglutide was launched in 2010, dulaglutide in 2014, and tirzepatide in 2022 (Figure 2a). Over the past 5 years, semaglutide has accounted for the majority of reported cases. By gender, the incidence of cholecystitis and cholelithiasis among female patients using GLP-1 RAs was 58.72%, significantly higher than the 37.07% observed in male patients (Figure 2b). However, dulaglutide exhibited a nearly equal male-to-female ratio. Regarding age, the 45-64 age group was the most affected, accounting for 33.84% of all cases (Figure 2c). Notably, dulaglutide was more concentrated in older patients, with 32.79% of cases involving those aged ≥65 years. Consumers and physicians were the main reporters of these AEs, accounting for 43.41% and 39.57% of submissions, respectively (Figure 2d). In terms of geographic distribution, the majority of reports came from the United States (67.80%), with reports from other countries contributing less than 5% (Figure 2e). Notably, 92.01% of reports were classified as severe. Hospitalization was the most common outcome, occurring in 49.93% of cases, followed by other serious conditions such as life-threatening events and disability (Figure 2f).

### 3.2 Distribution of AE onset time

**FIGURE 3:**
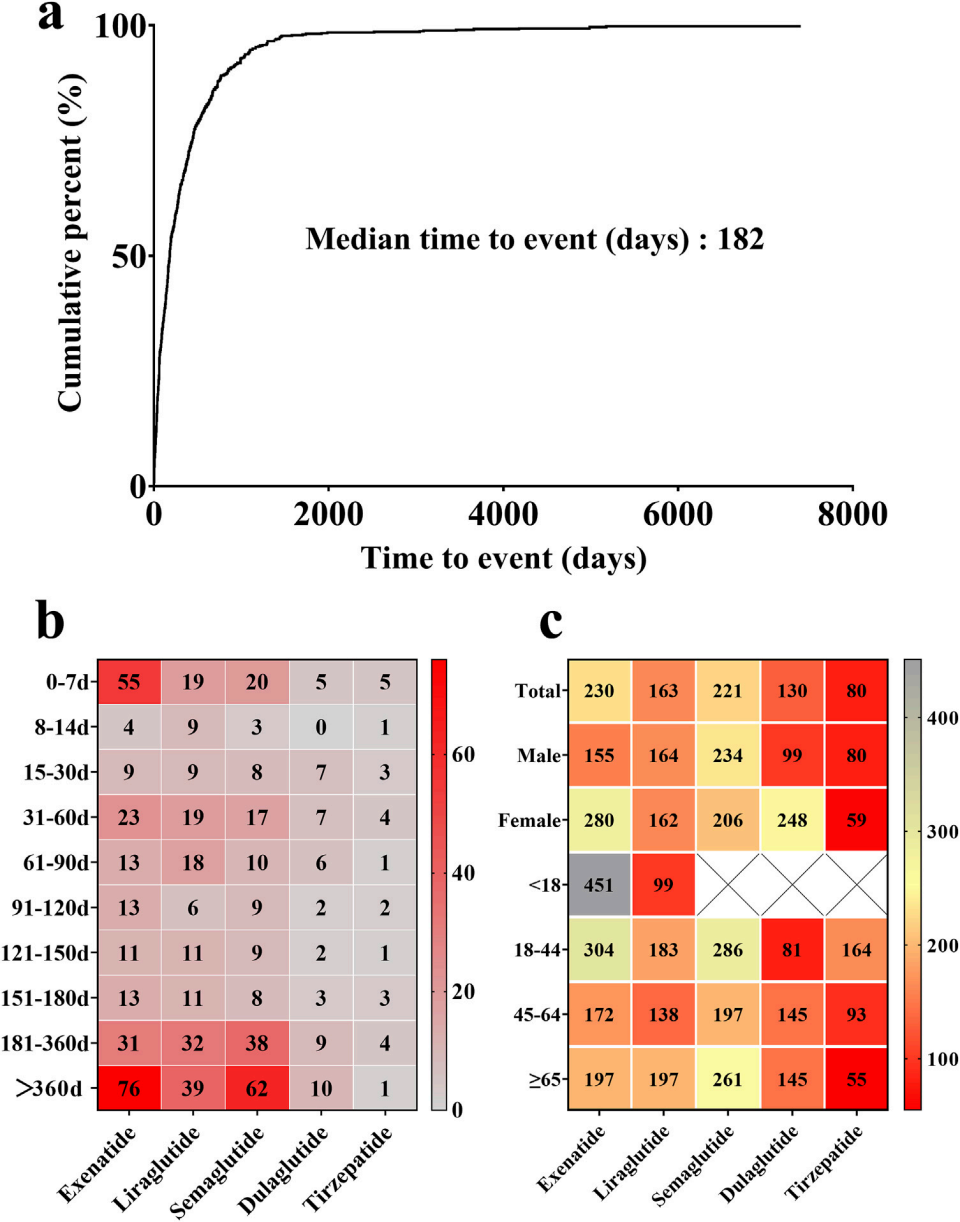
**(a)** Median onset time of GLP-1 RA-induced cholecystitis and cholelithiasis. **(b)** Proportional distribution of GLP-1 RA-induced cholecystitis and cholelithiasis incidence across various time intervals. Red blocks indicate significant associations between cholecystitis and cholelithiasis and specific time periods, while grey blocks denote weak associations. **(c)** Median onset time of GLP-1 RA-induced cholecystitis and cholelithiasis across different genders and age groups. Red blocks indicate shorter median onset times, while grey blocks denote longer median onset times.

As depicted in Figure 3a, the median onset time of GLP-1 RA-induced cholecystitis and cholelithiasis was 182 days. For exenatide, liraglutide, and semaglutide, cholecystitis and cholelithiasis typically occurred after 360 days (Figure 3b). However, it is noteworthy that a considerable proportion of patients developed these conditions within 7 days of initiating exenatide treatment. Tirzepatide exhibited the shortest median onset time for cholecystitis and cholelithiasis, at 80 days, while exenatide had the longest median time, at 230 days. Overall, the median time to onset differed significantly among the various GLP-1 RAs (*P* < 0.0001). Gender differences were also observed in the onset times for specific drugs (Figure 3c). Female patients experienced a longer median onset time for cholecystitis and cholelithiasis with exenatide and dulaglutide compared to male patients, with differences of 125 and 149 days, respectively. In contrast, male patients exhibited a longer median onset time for these conditions when using semaglutide and tirzepatide. For liraglutide, the median onset time for cholecystitis and cholelithiasis was similar in both male and female patients, with respective values of 164 and 162 days.

By patient age group, the median onset time for cholecystitis and cholelithiasis varied across different GLP-1 RAs. For exenatide and tirzepatide, the median onset time generally decreased with age, although exenatide showed some fluctuations. Conversely, dulaglutide exhibited an increasing trend in median onset time as age progressed. Notably, exenatide displayed the longest median onset time for cholecystitis and cholelithiasis in patients under 18 years old, at 451 days, while tirzepatide had the shortest median onset time in patients over 65 years old, at 55 days.

### 3.3 Proportional distribution of drugs in AE reports

**FIGURE 4:**
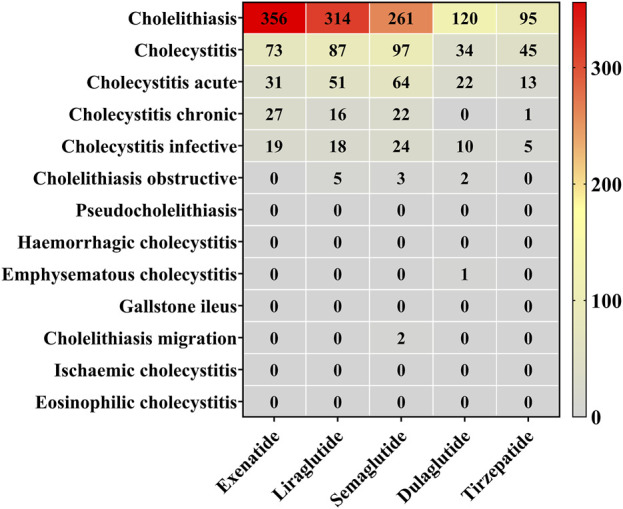
Reporting proportions at the HLT and PT levels. Red blocks indicate significant associations between cholecystitis and cholelithiasis and their corresponding PT, while grey blocks denote non-significant associations.

Based on the frequency of AE reports, we summarized the distribution of reports for GLP-1 RAs at both the HLT and PT levels (Figure 4). Semaglutide was associated with the highest number of PTs. Cholelithiasis (PT) was the most frequently reported AE across all drugs, with exenatide (n = 356, 70.36%) exhibiting the highest proportion, followed by liraglutide (n = 314, 63.95%), dulaglutide (n = 120, 63.49%), tirzepatide (n = 95, 59.75%), and semaglutide (n = 261, 55.18%). Reports of cholecystitis (PT) and cholecystitis acute (PT) were also frequent, with semaglutide contributing the most reports (n = 97 and n = 64, respectively). In contrast, other PTs were less frequently reported, and less common conditions, such as cholelithiasis obstructive and pseudocholelithiasis, had very few or no associated reports.

### 3.4 AE signal detection results

**FIGURE 5:**
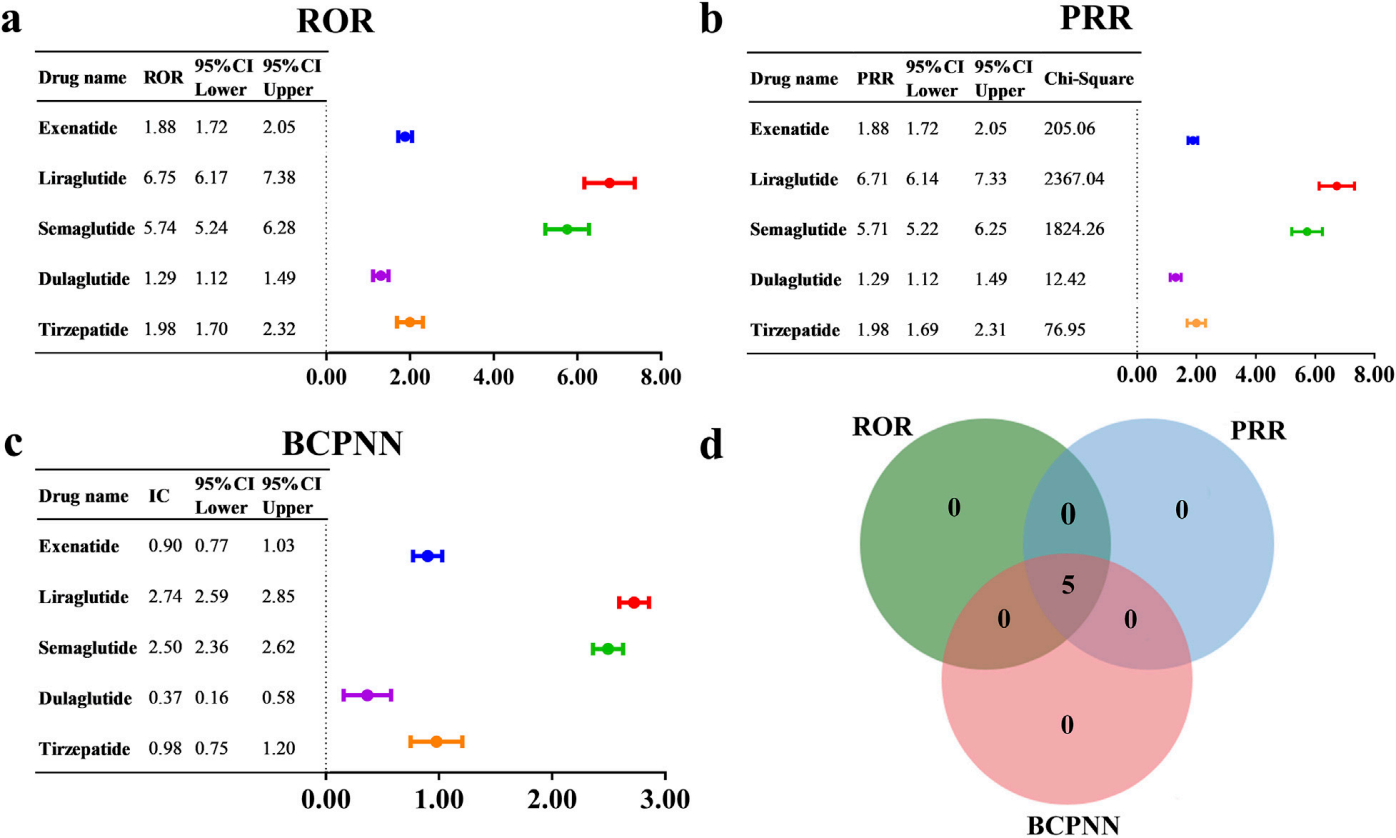
AE signal detection results for GLP-1 RAs associated with cholecystitis and cholelithiasis, based on **(a)** ROR, **(b)** PRR, and **(c)** BCPNN. **(d)** The number of positive drugs identified by the ROR, PRR, and BCPNN methods.

To evaluate the potential risk of cholecystitis and cholelithiasis induced by GLP-1 RAs, we utilized three methods, including ROR, PRR, and BCPNN, to detect and conduct a comprehensive analysis of AE signals. The results are presented in Figures 5a–c. Specifically, the following signals were detected: exenatide (N = 506, ROR = 1.88, 95% CI = 1.72–2.05, PRR = 1.88, χ^2^ = 205.06, IC = 0.90, IC025 = 0.77), liraglutide (N = 491, ROR = 6.75, 95% CI = 6.17–7.38, PRR = 6.71, χ^2^ = 2367.04, IC = 2.74, IC025 = 2.59), semaglutide (N = 473, ROR = 5.74, 95% CI = 5.24–6.28, PRR = 5.71, χ^2^ = 1824.26, IC = 2.50, IC025 = 2.36), dulaglutide (N = 189, ROR = 1.29, 95% CI = 1.12–1.49, PRR = 1.29, χ^2^ = 12.42, IC = 0.37, IC025 = 0.16), tirzepatide (N = 159, ROR = 1.98, 95% CI = 1.70–2.32, PRR = 1.98, χ^2^ = 76.95, IC = 0.98, IC025 = 0.75). The signal detection results indicated that GLP-1 RAs displayed positive signals across all three detection methods (Figure 5D). Notably, the overall GLP-1 RA group also exhibited a positive signal (N = 1,829, ROR = 2.86, 95% CI = 2.73–3.00, PRR = 2.86, χ^2^ = 2132.34, IC = 1.48, IC025 = 1.41). Detailed results are provided in Supplementary Table S1. Furthermore, a review of the drug instructions for GLP-1 RAs revealed that only the instructions for semaglutide do not list cholecystitis and cholelithiasis as an AE or mention them in the precautions.

## 4 Discussion

This study employed the FAERS database and applied three disproportionate analysis methods—ROR, PRR, and BCPNN—to quantify the association strength between GLP-1 RAs and cholecystitis and cholelithiasis. Higher signal detection values indicate stronger associations, reflecting an elevated risk of developing these conditions with GLP-1 RAs. This study also explored variations in the onset time of cholecystitis and cholelithiasis following GLP-1 RA treatment. Furthermore, we investigated the distribution of GLP-1 RAs at both the HLT and PT levels and conducted signal detection analyses.

This study observed a dynamic trend in the number of reports related to GLP-1 RA-induced cholecystitis and cholelithiasis, corresponding to the launch of newer drugs. Since 2021, the volume of reports has been steadily increasing. Notably, the number of reports in the first half of 2024 has nearly reached the total for 2023, suggesting a substantial rise in cases of GLP-1 RA-induced cholecystitis and cholelithiasis. In terms of gender, the overall rate of cholecystitis and cholelithiasis was notably higher in female patients than in male patients, suggesting the need for heightened caution when prescribing these drugs to female patients. Regarding age, the incidence of GLP-1 RA-induced cholecystitis and cholelithiasis varied among different age groups. The findings indicate that increased vigilance is warranted when prescribing exenatide, liraglutide, semaglutide, and tirzepatide to patients aged 45-64, and dulaglutide to patients aged 65 years and older.

The median onset times for exenatide and dulaglutide to induce cholecystitis and cholelithiasis exhibited significant gender differences, with females displaying a notably longer onset time than males. Conversely, for tirzepatide, the onset time was shorter in females than in males. This indicates that healthcare providers should consider gender differences when monitoring for cholecystitis and cholelithiasis in T2DM or obese patients using these drugs. Furthermore, increased vigilance is needed for liraglutide in patients under 18 years, dulaglutide in the 18-44 age group, and tirzepatide in patients aged 45 and above, as these age groups show shorter median times for the occurrence of cholecystitis and cholelithiasis.

The majority of studies indicate that GLP-1 RAs are associated with an increased risk of gallbladder or biliary tract diseases (Wang et al., 2022; Woronow et al., 2022; Gameil et al., 2024; Pratley et al., 2024; Wang et al., 2024; Yang et al., 2024), such as cholecystitis and cholelithiasis (Nreu et al., 2020; Zeng et al., 2023). A meta-analysis has demonstrated a significant association between GLP-1 RAs and an increased risk of acute cholecystitis, with a pooled risk ratio of 1.51 (95% CI: 1.08–2.09) (Wang et al., 2024). High doses, prolonged use, and rapid weight loss have been identified as factors contributing to this increased risk (He et al., 2022; Yang et al., 2024). For instance, a systematic review and meta-analysis of a randomized clinical trial found that weight loss following GLP-1 RA therapy was significantly associated with an increased risk of gallbladder or biliary disease, with an odds ratio of 1.361 (95% CI: 1.147–1.614; P < 0.001; I^2^ = 3.5%) (Yang et al., 2024). Additionally, several systematic reviews have indicated that semaglutide is primarily associated with an increased risk of cholelithiasis (Smits and Van Raalte, 2021; Bensignor et al., 2024), with reported cases documenting semaglutide-induced cholelithiasis (Eckard et al., 2024). Moreover, a systematic review and meta-analysis of a randomized controlled trial found that semaglutide was associated with an increased risk of gallbladder disease (odds ratio: 1.26, p = 0.010) and cholelithiasis (odds ratio: 2.06, p = 0.04) (Kommu and Berg, 2024). A strong association between semaglutide and acute cholecystitis has been identified in several systematic reviews and pharmacovigilance analyses (Du et al., 2024; Kommu and Berg, 2024; Xiong et al., 2024; Kushner et al., 2025). Notably, the oral formulation of semaglutide induces a higher number of gallbladder-related AEs compared to the subcutaneous formulation (Aroda et al., 2023). Moreover, in obese patients achieving a 10% weight loss, semaglutide demonstrated the greatest clinical benefits, while the incidence of adverse drug reactions increased progressively with further weight loss (Moll et al., 2024). Liraglutide has also been linked to an increased risk of gallbladder diseases (Smits and Van Raalte, 2021). In a randomized, head-to-head, placebo-controlled trial, a higher incidence of cholelithiasis was observed in the liraglutide treatment group compared to placebo (Lundgren et al., 2021). Additionally, the LEADER randomized trial demonstrated a significantly increased risk of acute gallbladder or biliary disease in the liraglutide group compared to the placebo group, with a hazard ratio of 1.60 (95% CI: 1.23–2.09; P < 0.001) (Nauck et al., 2019). Compared with dulaglutide, liraglutide exhibits a stronger association with gallbladder and biliary tract diseases (Dong et al., 2022). Tirzepatide appears to be relatively safer among the GLP-1 RAs, with a lower risk of gallbladder or biliary tract diseases (Tang et al., 2022; Mishra et al., 2023; Kamrul-Hasan et al., 2024). However, it is significantly associated with the composite of gallbladder or biliary diseases (Zeng et al., 2023). Additionally, a systematic review and meta-analysis of randomized controlled trials indicated a slightly increased incidence of cholecystitis in the tirzepatide group compared to the placebo group (Cai et al., 2024). These findings further support the association between GLP-1 RAs and an elevated risk of cholecystitis and cholelithiasis.

GLP-1 RAs may induce cholecystitis and cholelithiasis through multiple mechanisms, including altering bile composition by increasing cholesterol secretion into bile. This can elevate the bile cholesterol saturation index, consequently promoting the formation of cholesterol stones, affecting gallbladder function, and disrupting bile acid metabolism (Nreu et al., 2020). Rapid weight loss, a common effect of GLP-1 RA use, is a significant factor in the development of cholelithiasis. It can result in cholesterol supersaturation, metabolic disturbances, and alterations in bile composition. Additionally, rapid weight loss reduces the secretion of cholecystokinin, which diminishes gallbladder motility and delays gallbladder emptying, ultimately leading to the formation of cholesterol crystals and cholelithiasis (Nreu et al., 2020; Yang et al., 2024). Long-term use of GLP-1 RAs may chronically overstimulate GLP-1 receptors, leading to alterations in bile composition and motility, which can eventually result in cholelithiasis (Aroda et al., 2023; Zeng et al., 2023). Moreover, GLP-1 RAs can delay postprandial gallbladder refilling or induce changes in gallbladder motility, contributing to the formation of cholelithiasis or inflammatory changes in the gallbladder (Yang et al., 2024).

We further explored potential mechanistic differences among various GLP-1 RAs in their associations with cholecystitis and cholelithiasis. Long-acting GLP-1 RAs, such as semaglutide and dulaglutide, may exert a more pronounced inhibitory effect on gallbladder contraction due to their extended half-lives and sustained pharmacological profiles, thereby increasing the risk of gallbladder-related AEs (Trujillo et al., 2021). In contrast, short-acting agents like exenatide, with a shorter duration of action, may have a relatively milder impact on gallbladder function (Trujillo et al., 2021), which aligns with our findings. Additionally, liraglutide has demonstrated notable efficacy in reducing both HbA1c levels and body weight, but it is also associated with a higher incidence of gastrointestinal AEs, which may indirectly impair gallbladder function (Ma et al., 2023). Differences in dosing frequency and patient adherence across various GLP-1 RAs may also influence their impact on gallbladder health. For instance, the once-weekly administration of dulaglutide may be more acceptable to patients, potentially improving adherence and reducing the risk of gallbladder dysfunction related to inconsistent treatment (Tsapas et al., 2020). Therefore, for high-risk populations (particularly older women), close monitoring for cholecystitis and cholelithiasis is recommended during GLP-1 RA therapy. If patients present with symptoms such as upper abdominal pain, nausea, or vomiting, prompt imaging evaluation (e.g., gallbladder ultrasound) should be conducted to confirm the diagnosis. Moreover, extra caution is warranted in patients receiving higher doses, undergoing long-term treatment, or experiencing rapid weight loss, as these factors may further elevate the risk of developing cholecystitis or cholelithiasis.

Although this study primarily focused on reports of GLP-1 RA-induced cholecystitis and cholelithiasis, we also applied the shrinkage measure method to analyze 147 concomitant medications used alongside GLP-1 RAs for signal detection (Noguchi et al., 2020a; Noguchi et al., 2020b). Detailed results are presented in Supplementary Table S2. Only two drug combinations were reported more than ten times: exenatide with sitagliptin (n = 17, Ω = 0.97, Ω025 = 0.29) and exenatide with metformin (n = 21, Ω = 0.67, Ω025 = 0.05). Although both combinations yielded Ω025 values greater than zero, indicating positive signals, the vast majority (140 out of 147) exhibited negative signals. The limited sample size may compromise the performance of signal detection algorithms, making it challenging to distinguish true safety signals from background noise. Therefore, due to the small number of reports and the statistical limitations, we currently lack sufficient evidence to explore potential associations between GLP-1 RAs and concomitant medications in the development of cholecystitis and cholelithiasis.

Despite the comprehensive investigation of the risk of GLP-1 RA-induced cholecystitis and cholelithiasis using the FAERS database, several limitations should be acknowledged. First, reports in the FAERS database may be incomplete, with the majority originating from the United States. Reporting behaviors can vary significantly across patient populations, such as by age, gender, and educational background, which may introduce geographic and demographic biases. Second, differences in drug approval dates and the clinical usage frequency of individual GLP-1 RAs may influence the number of reported cases. Third, patients with T2DM often require long-term or lifelong pharmacotherapy, and comorbidities such as obesity and metabolic disorders may further impact the risk of developing cholecystitis and cholelithiasis. Therefore, further studies are needed to elucidate the potential interactions of comorbidities and concomitant medications in order to better assess and manage the risk of gallbladder-related AEs. Finally, although large-scale pharmacovigilance databases like FAERS are valuable for identifying statistical associations between GLP-1 RAs and AEs, they have inherent limitations that preclude the calculation of incidence rates or absolute risks and do not allow for the establishment of causality. Nonetheless, certain strategies may help overcome these limitations. For instance, conducting prospective cohort studies and multicenter collaborative research can help reduce underreporting and reporting bias. Furthermore, integrating FAERS data with prescription databases—particularly by leveraging big data technologies and electronic health records—can provide more detailed information on drug exposure. The combined use of these approaches may progressively overcome the FAERS limitations in future research, ultimately enhancing the accuracy of adverse drug reaction surveillance.

## 5 Conclusion

This study systematically analyzed data from the FAERS database, covering from Q1 2004 to Q2 2024, to investigate reports of cholecystitis and cholelithiasis in which GLP-1 RAs were identified as the primary suspect drug. AE reports related to these conditions were identified for five commonly used GLP-1 RAs: exenatide, liraglutide, semaglutide, dulaglutide, and tirzepatide. The results revealed that GLP-1 RA-induced cholecystitis and cholelithiasis predominantly occur in patients aged 45 and older, with a notably higher incidence observed in female patients. The overall median time to onset for GLP-1 RA-induced these AEs was 182 days, with tirzepatide demonstrating the shortest median onset time of 80 days. Additionally, variations in the onset time were observed among different medications, influenced by gender and age group. Our findings provide valuable insights for identifying and mitigating GLP-1 RA-induced cholecystitis and cholelithiasis in real-world settings, aiming to optimize clinical practice and enhance treatment safety.

## Data Availability

The datasets presented in this study can be found in online repositories. The names of the repository/repositories and accession number(s) can be found in the article/Supplementary Material.
