# Supplementary material for: Glucagon-like peptide-1 receptor agonist-induced cholecystitis and cholelithiasis: a real-world pharmacovigilance analysis using the FAERS database
Source: Front Pharmacol. 2025 Jul 8;16:1557691. doi: 10.3389/fphar.2025.1557691 (PMC12279493; doi:10.3389/fphar.2025.1557691)
Supplement: Supplementary file 2 [file Table1.docx]

**Supplementary material for**

**Glucagon-Like Peptide-1 Receptor Agonist-Induced Cholecystitis and Cholelithiasis: A Real-World Pharmacovigilance Analysis Using the FAERS Database**

**Table S1**. AE signal detection results for GLP-1 RAs associated with cholecystitis and cholelithiasis (HLT).

| **Drug name** | **No. of reports** | **ROR (95% CI)** | **Signal** | **PRR (95% CI)** | **χ^2^** | **Signal** | **IC (95% CI)** | **Signal** |
| --- | --- | --- | --- | --- | --- | --- | --- | --- |
| GLP-1 RAs | 1829 | 2.86 (2.73-3.00) | Y | 2.86 (2.73-2.99) | 2132.34 | Y | 1.48 (1.41-1.55) | Y |
| Exenatide | 506 | 1.88 (1.72-2.05) | Y | 1.88 (1.72-2.05) | 205.06 | Y | 0.90 (0.77-1.03) | Y |
| Liraglutide | 491 | 6.75 (6.17-7.38) | Y | 6.71 (6.14-7.33) | 2367.04 | Y | 2.74 (2.59-2.85) | Y |
| Semaglutide | 473 | 5.74 (5.24-6.28) | Y | 5.71 (5.22-6.25) | 1824.26 | Y | 2.50 (2.36-2.62) | Y |
| Dulaglutide | 189 | 1.29 (1.12-1.49) | Y | 1.29 (1.12-1.49) | 12.42 | Y | 0.37 (0.16-0.58) | Y |
| Tirzepatide | 159 | 1.98 (1.70-2.32) | Y | 1.98 (1.69-2.31) | 76.95 | Y | 0.98 (0.75-1.20) | Y |

GLP-1 RAs include albiglutide and lixisenatide. Signal: Y: Yes, positive signal. N: No, negative signal.
